# Do Social Relationships Influence Moral Judgment? A Cross-Cultural Examination

**DOI:** 10.3390/bs15081097

**Published:** 2025-08-12

**Authors:** Lina Ding, Lei Fu, Kai Li, Feng Yu

**Affiliations:** 1 School of Philosophy, Wuhan University, Wuhan 430072, China; dinglina@whu.edu.cn; 2 Department of Psychology, Wuhan University, Wuhan 430072, China; fulei1075@whu.edu.cn

**Keywords:** social relationships, moral judgment, culture

## Abstract

This study examines whether social relationships influence moral judgment across different types of moral violations and cultural contexts. Drawing on Relationship Regulation Theory, which outlines four relational models—communal sharing, authority ranking, equality matching, and market pricing—we investigate whether moral evaluations vary depending on the relationship between the actor and the victim. Unlike previous research that primarily adopts a third-party perspective, this study uses a first-person approach, focusing on judgments made by individuals directly involved in the moral interaction. Three empirical studies were conducted: Study 1 tests the influence of social relationships on moral judgment using Chinese participants; Study 2 explores how moral judgments differ across various moral domains in relational contexts; and Study 3 compares Chinese and American participants to assess cross-cultural differences in the impact of social relationships on moral evaluation. Across all three studies, the results consistently show that social relationships significantly affect moral judgment, supporting the view that moral evaluations are shaped not only by the nature of the act but also by the relational context in which it occurs.

## 1. Introduction

Morality has long been a central focus of psychological research, examining how people form judgments about right and wrong in response to harm, virtue, and social norms (Gray et al., 2012). Early work demonstrated that the perceived severity of an action’s consequences and individuals’ religious or ideological commitments both shape moral evaluations (Graham et al., 2009). Building on these findings, Haidt’s Social Intuitionist Model argues that rapid, emotion-driven intuitions primarily underlie moral judgments, with conscious reasoning serving mainly to justify those intuitions (Haidt, 2001). Complementing this view, Greene’s dual-process theory posits that fast, affective processes favor deontological judgments, while slower, deliberative reasoning supports utilitarian choices; neuroimaging studies using dilemmas like the trolley problem confirm distinct brain activations for each pathway (Greene et al., 2001; Greene, 2014). More recent empirical studies have further illuminated the role of emotion in moral reasoning. For example, a study of clinical ethics consultations found that professionals frequently experience strong frustration, sadness, and guilt during deliberations, which, in turn, affect both their in-the-moment judgments and retrospective evaluations (Dahò, 2025). A neurocognitive model of perceptual decision-making on emotional signals reveals that tasks demand recruit distinct neural pathways—particularly the left amygdala—for verbal versus nonverbal emotion recognition (Dricu & Frühholz, 2020). These findings suggest that emotional responses are not peripheral but central to moral cognition, especially in real-world contexts.

In parallel, recent research has increasingly emphasized that moral judgment standards are not solely based on perceived severity or emotional reactions—they are also shaped by the social context in which the act occurs (Earp et al., 2021; Simpson et al., 2016). For example, Simpson et al. (2016), drawing on Relational Models Theory and Moral Foundations Theory, demonstrated that the type of relationship between the actor and the recipient significantly influences third-party evaluations of moral wrongness. Behaviors deemed acceptable within intimate relationships may be harshly condemned in hierarchical or exchange-based contexts. Building on this perspective, Earp et al. (2021) proposed the Relational Norms Model, which systematically explains how the cooperative functions expected within different social relationships—such as care, reciprocity, hierarchy, or mating—shape moral evaluations. Across two large-scale preregistered studies, they found that individuals are more likely to judge a behavior as morally wrong when it violates the normative expectations associated with a specific relationship type. This model outperforms traditional predictors such as genetic relatedness or interpersonal closeness, highlighting the central role of functional expectations in moral cognition.

However, prior studies have primarily relied on third-party perspectives, where observers evaluate the morality of others’ actions (Reader & Hughes, 2020). This leaves open the question of whether individuals directly involved in moral situations—particularly victims—judge moral violations differently depending on their relationship with the actor. Emotional responses may be especially salient in such first-person contexts, where personal involvement intensifies moral sensitivity. To address this gap, the present study adopts a first-person perspective, focusing on moral judgments made by actors or victims themselves. This approach offers a more ecologically valid understanding of how relational context influences moral evaluation from within the interaction. Additionally, moral judgment standards vary across cultures, with notable differences between Eastern and Western societies. This study therefore examines the impact of social relationships on moral judgment in both Chinese and Western cultural contexts, emphasizing cases where the moral judge is the victim rather than an uninvolved observer. While the present research does not directly investigate emotional mechanisms, the role of emotion in shaping first-person moral evaluations will be considered in the Section 6.

## 2. Theoretical Background and Research Hypotheses

### 2.1. Moral Judgment and Relationship Regulation Theory

Morality is influenced by social relationships (Earp et al., 2021). The same actions are not judged as immoral when occurring between parents and children but are deemed immoral by most when occurring between superiors and subordinates (Simpson et al., 2016). Some scholars believe that tradition cannot explain why people criticize transactional behaviors, because morally critical transactions are not beneficial (Earp et al., 2021). What are the benefits of making ethical decisions? Previous studies have found that alliances can be highly disruptive, leading to personal conflicts and even ethnic wars (DeScioli, 2023). People judge others as morally worse when they abuse their families compared with when they abuse friends or coworkers (Earp et al., 2021). People believe it is permissible for their parents to make cruel remarks about their weight and appearance, whereas it is perceived as taboo for their colleagues to make similar comments. People who help strangers are judged as more morally good, whereas people who help close relatives are not judged to be morally good (Graham et al., 2011). Not helping family members may be judged as gravely wrong, but it is considered acceptable when this occurs between a customer and seller (Simpson et al., 2016). Relational context refers to the type or nature of the relationship between the person committing the act (the actor) and the person affected (the victim); for instance, whether they are strangers, friends, or family. This context influences how people judge the morality of an action. The relational context is a strong predictor of wrongness judgments of loyalty, respect, and purity violations (Dricu & Frühholz, 2020). Third parties refer to individuals who are not directly involved in the moral event itself. That is, they are neither the actor nor the victim but instead are external observers who evaluate the situation. Much of the previous research has focused on how these third-party observers make moral judgments in hypothetical or observed scenarios, rather than when the moral event directly involves the participants’ own relationships. However, there is still a lack of empirical research on why social relationships influence moral judgments when moral events occur between the actor and the victim.

According to Relationship Regulation Theory, there are four relational models: communal sharing, authority ranking, equality matching, and market pricing (Fiske, 1992). Corresponding to these four relationship types are four moral motivations: (1) unity, which encourages an “all for one, one for all” mentality; (2) hierarchy, which functions to maintain ordinal social rank; (3) equality, which ensures reciprocity and balanced treatment, responsibilities, and rights; and (4) proportionality, which guides moral action and judgment via ratio-like considerations of what is proportionately or rationally appropriate (Rai & Fiske, 2011). People think that different moral standards need to be followed under different social relationships. For instance, when performing a task with a stranger and the workloads vary, not allocating rewards in accordance with the proportion of contributions is unethical. But when performing a task with parents or siblings, not distributing rewards based on the contribution ratio is not regarded as unethical. Moral motivation can be used to regulate and maintain social relations, which influence moral judgments (Zakharin & Bates, 2023). However, few empirical studies have investigated the relationships among relational model types and moral motivations. Thus, we hypothesize the following:

**H1.**  *Moral judgments are shaped by social relationships; the perceived immorality of an action can vary depending on whether it occurs within a parent–child, superior–subordinate, colleague–colleague, or salesperson–customer relationship.*

### 2.2. Cultural Differences Between the East and the West

The lay concept of “immoral behavior” is different in Eastern and Western cultures (Buchtel et al., 2015; Buchtel et al., 2018). Moral judgments are also influenced by culture (Cohen, 2009; Shweder, 1997). Closeness and status are identified as the basic dimensions that define one’s moral duties and responsibilities vis-à-vis others in the culture of Confucianism (Hwang, 2012). For example, research on individualism–collectivism also found that Western, educated, industrialized, rich, and democratic (WEIRD) cultures are more apt to endorse moral codes emphasizing individual rights and independence, whereas non-WEIRD cultures tend to moralize duty-based communal obligations and spiritual purity more strongly (Haidt et al., 1993; Hofstede, 1984). In this study, we focus on the effects of the individualism–collectivism value. Individualism–collectivism is a cultural dimension that measures “the degree of societal interdependence among individuals” (Oyserman et al., 2002). Collectivism promotes the relevance of a group and society over individual objectives (Oyserman et al., 2002). In this study, we hypothesized H2:

**H2.**  *The influence of social relationships on moral judgments is also affected by the cultural differences between the East and the West.*

Therefore, this study examines whether social relationships influence moral judgment and the differences between Chinese and Americans in this context. We extend prior work by conducting three studies using different methods. Study 1 examines the impact of social relationships on moral judgment by experimenting with a non-WEIRD sample. Study 2 explores how moral judgments differ across various moral domains in relational contexts. Study 3 investigates the moral judgment differences between Chinese and Americans. Across these three studies, we clarify the relationships between social relationships and moral judgment.

## 3. Study 1

Study 1 examines the influence of social relationships on moral judgment in non-WEIRD cultures using a between-subjects experimental design.

### 3.1. Method

#### 3.1.1. Participants

To obtain a medium power test (effect size f = 0.25 in an ANOVA analysis), a G∗Power analysis suggested that a total sample size of 242 participants would be needed to obtain a power of 0.95 (Faul et al., 2009). However, because we did not know the “true effect,” we oversampled in Study 1. Therefore, we recruited 454 participants (42.7% male, N = 194; 57.3% female, N = 260; 88.8% college, 7% master’s degree, and 4.2% high school level or lower; 98% were Han Chinese and 2% belonged to other ethnic groups; all participants identified as East Asian). Participants were aged between 20 and 50 years (M = 28.86; SD = 4.46) and were recruited through the online platform Credamo (https://www.credamo.com/).

All participants were randomly assigned to one of four social relationship conditions (parents vs. superiors vs. officemates vs. salespersons), after which they were asked to rate the moral wrongness of eight vignettes. An example of a scenario is: “You and your parents/superiors/colleagues/salespersons perform work, and you do 80% of the work on your own. After the work is completed, you receive payment for the work completed. Your parents/superiors/colleagues/salespeople do not share 80% of the payment, but instead they give you half of it.”.

#### 3.1.2. Materials and Procedure

##### Relational Contexts

We chose the parent–child, superior–subordinate, colleague–colleague, and salesperson–customer relationships to represent four relational contexts. These four relationships were typically discussed in the pertinent literature and used in the study of relational contexts and moral judgment (Earp et al., 2021; Fiske, 1992).

##### Moral Judgment

Participants were requested to assume that they were involved in scenarios that may occur in daily life. After reading each scenario, participants answered questions by assessing how immoral or moral the agent was (1 = not immoral at all; 7 = very immoral). The higher the score, the more immoral participants perceived the behavior to be. We varied the relationship between the victim and the actor (relationship: parents vs. superiors vs. colleagues vs. salesperson) (the Cronbach’s alpha coefficient for these was 0.75, 0.86, 0.85, 0.82).

#### 3.1.3. Data Analysis

The present study employed a single-factor experimental design with four levels. Accordingly, a one-way analysis of variance (ANOVA) was conducted to examine group differences, using SPSS version 27 for statistical analysis. Prior to performing the ANOVA, the data were assessed for normality. The results indicated that the assumption of normal distribution was met, thereby justifying the use of parametric statistical procedures.

### 3.2. Results

#### Moral Judgment

We conducted a one-way analysis of variance (ANOVA) with relational context (parents vs. superiors vs. colleagues vs. salesperson) as a between-subjects independent variable and moral judgment as the dependent variable. As predicted, there was a significant effect of relational context on moral judgment(F(3, 450) = 8.73; *p* < 0.001).

Post hoc comparisons using Tukey’s HSD test revealed that moral transgressions involving parents were perceived as the least immoral, whereas those involving a salesperson were judged as the most immoral. The difference between these two groups was statistically significant (*p* < 0.001; 95% CI = [−0.97, −0.30]). No significant differences were found among the other relational contexts.

The means and standard deviations of moral judgments across the four relational contexts are presented in Table 1.

## 4. Study 2

In Study 1, we provided evidence that social relationships influence moral judgment using eight scenarios. To enhance the robustness of our findings and to expand the range of immoral scenarios, we conducted Study 2, which included five different types of moral transgressions (Care, Fairness, Loyalty, Respect, and Purity). Furthermore, to demonstrate the generalizability of the phenomenon observed in Study 1, we employed moral scenarios derived from Moral Foundations Theory.

### 4.1. Methods

#### 4.1.1. Participants

To obtain a medium power test (effect size f = 0.25 in an ANOVA analysis), a G∗Power analysis suggested that a total sample size of 242 participants would be needed to obtain a power of 0.95 (Faul et al., 2009). The participants were 236 college students (32.6% male, N = 77; 67.4% female, N = 159; 97% Han Chinese and 3% belonging to other ethnic groups; all participants identified as East Asian), Participants were aged between 18 and 25 years (M = 19.25, SD = 1.10) and enrolled in general psychology courses.

#### 4.1.2. Materials and Procedure

Relational contexts: The same four relationships as in Study 1 were used.

Moral judgment: Moral violations were adapted from Simpson et al. (2016), consisting of 24 scenarios: 4 violations each representing the moral foundations of Care, Fairness, Loyalty, and Respect, and 8 violations representing Purity. Examples of each moral foundation are as follows: Care: Person A causes emotional harm to Person B but fails to offer an apology or seek reconciliation. Fairness: Person A purchases food with Person B but consumes more than an equal share. Loyalty: Person A does not support or stand up for Person B when others are unfairly rallying against them. Respect: Person A interrupts an important meeting that Person B is holding. Purity: Person A uses sexually lewd language when speaking to Person B. The participants were asked to rate the moral wrongness of each violation (for example, “your parents/superiors/colleagues/salesperson does X to Person B”). The mean reliability coefficients for each moral-foundation-set of violations (average alphas across all four relational contexts) were Care = 0.60, Fairness = 0.69, Loyalty = 0.68, Respect = 0.82, and Purity = 0.89. We included factors that may be associated with relational context and moral judgment. Participants answered questions on trusting others and relationships with others. Finally, participants rated moral wrongness (1 = not at all wrong; 7 = very wrong) for each of the 24 violations in each of the four relational contexts.

#### 4.1.3. Data Analysis

Given that the experiment employed a mixed factorial design, a mixed-design ANOVA was conducted to examine the effects of the independent variables. Specifically, relational contexts were manipulated between subjects, while moral foundations were manipulated within subjects. Statistical analyses were performed using SPSS version 27. Prior to conducting the ANOVA, the data were tested for normality and sphericity. The data met the assumption of normality, supporting the use of parametric procedures. Where the assumption of sphericity was violated, Greenhouse–Geisser corrections were applied to adjust the degrees of freedom accordingly.

### 4.2. Results

We conducted an analysis of variance with relational contexts (parents vs. superiors vs. colleagues vs. salespersons) and moral foundation (care vs. fairness vs. loyalty vs. respect vs. purity) as independent variables and moral judgment as the dependent variable.

**Figure 1:**
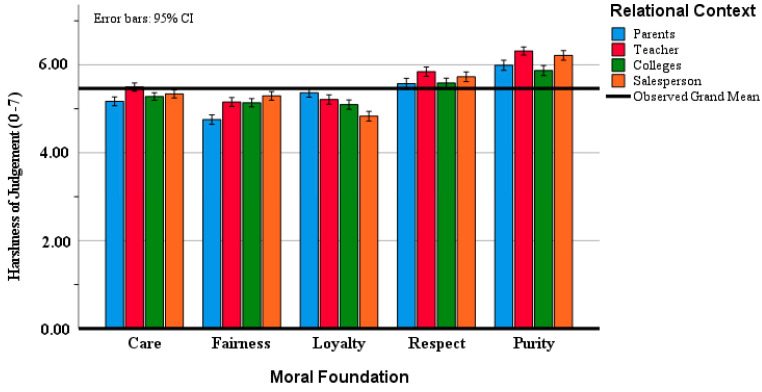
Harshness of moral judgment according to moral foundation and relational context. Note: Error bars represent standard error of the mean.

As predicted, the analysis revealed significant main effects as well as a significant interaction effect. There was an interaction between social context and moral foundation (F(12, 2424) = 33.00; *p* < 0.001; partial η^2^ = 0.14). The results also showed a main effect for the relational context. Post hoc comparisons using Tukey’s HSD test indicated that moral transgressions involving parents were perceived as the least immoral, whereas those involving a superior were judged as the most immoral. The difference between these two groups was statistically significant (*p* < 0.001; 95% CI = [−0.34, −0.13]), while no significant differences were found among the other relational contexts. Moral transgressions involving superiors were judged most harshly (M = 5.61; SD = 0.04), whereas those involving parents received the least severe judgment (M = 5.37; SD = 0.04) (F(3, 606) = 16.92; *p* < 0.001; partial η^2^ = 0.077). These results suggest that relational context significantly influences moral judgment. In addition, a Pearson correlation analysis was conducted to examine the relationships among moral judgment scores across the four relational conditions. The results indicated that moral judgments were positively correlated to a moderate degree, with correlation coefficients ranging from r = 0.518 to r = 0.719 (all with *p*-values < 0.05). Table 2 presents the means and standard deviations of moral judgments across the four relational contexts and five moral foundations, Figure 1 illustrates a more straightforward comparison of these results.

## 5. Study 3

The findings from Study 2 are consistent with our hypotheses. Moral judgments are also influenced by culture (Cohen, 2009; Shweder, 1997). It is possible that relational context differences in moral judgment emerged only in our Chinese sample. To address this concern, Study 3 tested the role of culture in a cross-cultural design.

### 5.1. Methods

#### 5.1.1. Participants

To obtain a medium power test (effect size f = 0.25 in an ANOVA analysis), a G∗Power analysis suggested that a total sample size of 242 participants would be needed to obtain a power of 0.95 (Oyserman et al., 2002). This study included 273 American participants from the Prolific survey company (46.9% male, N = 128; 53.1% female, N = 145; all from the United States). These participants were aged between 18 and 82 years (M = 43.72; SD = 14.28). The study also included 301 Chinese participants (54.2% male, N = 163; 45.8% female, N = 138; all from mainland China), who were aged between 18 and 64 years (M = 27.82; SD = 7.33).

#### 5.1.2. Materials and Procedure

Relational contexts: The same four relationships as in Study 1 were used.

Moral judgment: The scenarios were the same as in Study 2. Participants rated the moral wrongness (1 = extremely bad; 50 = neither bad nor good; 100 = extremely good) of each of the 24 violations in each of the four relational contexts.

#### 5.1.3. Data Analysis

Given that the experiment employed a two-factor between-subjects design, a factorial analysis of variance (two-way ANOVA) was conducted to examine the effects of the independent variables, using SPSS version 27 for statistical analysis. Prior to conducting the ANOVA, the data were tested for normality. The results indicated that the assumption of normal distribution was met, thereby supporting the use of parametric statistical procedures.

### 5.2. Results

#### Moral Judgment

Firstly, results from the interaction analysis indicated a significant interaction effect between social relationships and culture (F(3, 1716) = 51.88; *p* < 0.001; partial η^2^ = 0.08). Furthermore, both the main effect of social relationships and the main effect of culture reached statistical significance. To further examine the main effect of social relationships, a post hoc analysis using Tukey’s HSD test was conducted. The findings indicated that moral transgressions involving parents were perceived as the least immoral, whereas those involving a salesperson were judged as the most immoral. The difference between these two relational contexts was statistically significant (*p* < 0.001; 95% CI [−0.34, −0.13]), while no significant differences were observed among the remaining relational categories.

**Figure 2:**
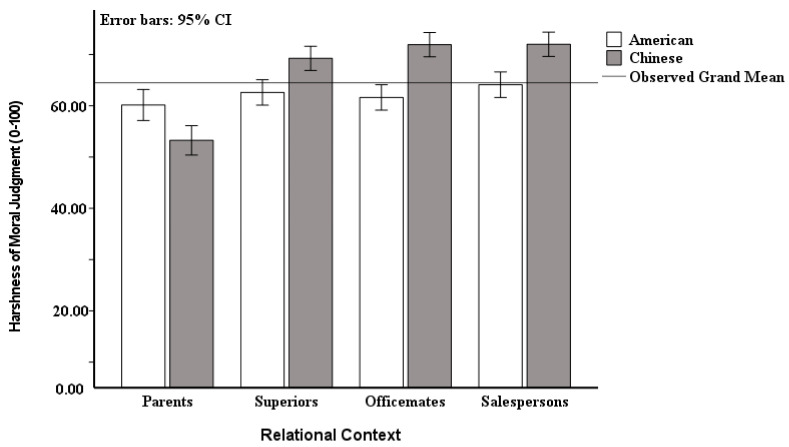
Differences in harshness of moral judgment between Chinese and American participants. Note: Error bars represent the standard error of the mean.

The Chinese have stricter moral judgment standards than those of Americans when moral violations occur between them and their superiors/officemates/salespersons, whereas Americans have stricter moral judgment standards than the Chinese when moral violations occur between them and their parents (Figure 2). Therefore, Chinese moral judgments are influenced more by social relations than those of Americans.

## 6. Discussion

This study clearly demonstrates that moral judgment varies across relational contexts, thereby supporting our hypothesis. This variation may reflect underlying cultural influences, consistent with emerging research suggesting that moral priorities differ across cultures (Buchtel et al., 2015; Buchtel et al., 2018). Our findings contribute to this growing body of work by highlighting how relationship context interacts with cultural background. Early philosophers expressed different views on the roles of emotion and reason in moral judgment. For example, Hume believed that emotion is the primary factor affecting moral judgment, whereas Kant believed that reason is the most important factor in moral judgments (Yu et al., 2011). Hume’s thoughts are biased toward consequentialism and utilitarianism. However, Kant insisted that morality is deontological (Denis, 2010; Johnson, 2011). This study extends previous work by using evidence-based methods to explore the role of social relationships in judgment.

### 6.1. The Relationship Between Social Relationships and Moral Judgment

As moral rules vary with changes in culture, situations, and interpersonal relationships, there are always controversies surrounding the same moral issues, given the vast and multidimensional nature of relationships and culture (Jin & Peng, 2021). Given the complexity and diversity of moral judgments, it is essential to consider various factors such as cultural norms and interpersonal dynamics. Recent studies have emphasized that moral evaluation is deeply influenced by social perception and cultural context (Alicke et al., 2015; Guglielmo, 2015). Research suggests that the moral evaluation of an action depends on the individuals involved (McManus et al., 2021). For instance, helping strangers is considered a moral act, whereas not helping strangers does not entail moral responsibility. Conversely, helping family members is not viewed as a moral act, but failing to help family members does incur moral responsibility (Marquette, 2020).

The concept of Relationship Regulation Theory is based on the framework proposed by Fiske (1991, 1992) and elaborated further in works by Rai and Fiske (2011, 2012), particularly in relation to moral judgment. These sources articulate that moral norms and expectations are largely shaped by the types of social relationships individuals inhabit. Recent studies have expanded this framework by examining how interpersonal emotion regulation and perceived social roles influence moral evaluations in relational contexts (Fiske, 1991; Rai & Fiske, 2011). According to this theory, there are four fundamental relational models through which people regulate their social interactions, each associated with distinct moral expectations: communal sharing—characterized by unity, shared identity, and mutual care (e.g., family or close friends); authority ranking—organized by hierarchy and respect, where superiors provide protection and subordinates offer deference (e.g., military, traditional family structures); equality matching—based on balance and reciprocity between individuals (e.g., peer relationships, turn-taking); and market pricing—driven by proportionality and cost–benefit calculations (e.g., business relationships, contracts). These relational models have been shown to activate different moral motives, thereby shaping individuals’ moral judgments (Fiske, 1991). For example, interpersonal emotion regulation within close relationships can lead to positively biased perceptions and influence moral expectations (Niven & López-Pérez, 2025). Morality, in this view, is the management of social relations as well as the regulation or rule of interaction between the subject and the object (Lemay et al., 2025). Therefore, different social relations need to abide by different norms, while things that do not involve relations do not involve morality (Ellemers, 2017).

However, different groups have different moral cognition. Therefore, the understanding of moral judgment should consider the social background. In the past, the paradigm of using abstract thinking to study morality had limitations and could not be used to understand moral conflicts in reality. Unlike previous theories, Relational Regulation Theory states that any behavior (including violence, unequal treatment, and indecent behavior) may be morally justified depending on the type of moral motivation and how social relations are structured. Recent research has shown that perceived social roles and external expectations significantly influence moral evaluations, especially in culturally diverse contexts (Dong et al., 2019). For example, this study found that the interpersonal relationship background significantly affects the third party’s judgment of moral violence and that the relationship between the two still holds after controlling for other factors that affect moral judgment (Mehdizadeh et al., 2021). Moreover, perceived stress has been found to alter moral schemas, shifting individuals toward more self-focused or consequence-sensitive reasoning, which further highlights the role of context in moral cognition (Chen et al., 2025). While participants distinguish between different types of social relationships when evaluating the immorality of an act, there is also a shared evaluative dimension underlying their moral judgments. In other words, individuals may apply a general moral sensitivity across contexts yet still modulate the severity of their judgments based on relational cues (Jordan, 2007; Katsarov, 2023; Marples, 2022). This relational modulation is supported by findings in interpersonal emotion regulation, where dependence on others for emotional support can shape moral expectations and evaluations within close relationships (Williams et al., 2018).

### 6.2. Cultural Variation in Social Relationships and Moral Judgment

Culture is a critical factor influencing moral judgment (Bentahila et al., 2021). Different societies face distinct types of social conflicts, prompting the development of moral norms tailored to those challenges. For instance, when promiscuity is morally sanctioned, individuals who pursue short-term relationships may face harsher condemnation than those seeking long-term commitments (Hurst, 2021; Wlodarski & Dunbar, 2015). These differences may reflect how moral rules are interpreted through the lens of relational motives (Mascolo et al., 2021; Rai & Fiske, 2011).

Our findings align with previous research suggesting that moral condemnation is modulated by relational proximity and cultural norms (Matsuo & Brown, 2022). In Eastern cultures, moral principles often emphasize collective harmony, such as loyalty and purity, whereas Western cultures prioritize individual rights and autonomy, such as fairness and care (Fiske, 1992; Garcia et al., 2013). Thus, cultural context plays a pivotal role in shaping how social relationships influence moral motivation. For example, in China, sexual behavior may be viewed as a collective moral issue, while in the United States, it is more likely to be considered a matter of personal choice (Garcia et al., 2013). Environmental conditions further shape cultural norms; resource-scarce environments tend to foster tight cultures with strict norms to manage social threats, whereas resource-abundant environments promote loose cultures with greater tolerance for deviant behavior (Gelfand et al., 2011; Graham et al., 2009). Consequently, permissive cultures exhibit more variability in how moral transgressions are perceived and punished (Chen-Xia et al., 2023). A large-scale study involving 348,660 participants found that women scored higher than men in moral dimensions such as care, fairness, and purity. Interestingly, gender differences in loyalty and authority were minimal but varied across cultures; in societies with greater gender equality, these differences in moral judgment between men and women were more pronounced (Atari et al., 2020).

Moreover, our findings suggest that moral evaluations are deeply relational. Whether an act is judged as moral or immoral often depends on the relationship between the actor and those affected. This implies that there may be no universally immoral motives or actions; rather, individuals tend to adopt moral reasoning that aligns with their relational and cultural interests. While this perspective highlights the contextual nature of morality, it also raises questions about the universality of moral principles and the potential for moral relativism in cross-cultural settings.

### 6.3. Practical Implications

Some practical implications and possible applications of our findings include the following: the design of educational programs aimed at fostering moral reasoning and emotional awareness; interventions that support ethical decision-making in clinical, organizational, and legal settings; cross-cultural communication strategies that account for differing emotional and cognitive moral frameworks. We believe these strengthen the applied relevance of our work and open avenues for interdisciplinary collaboration.

### 6.4. Limitations and Future Research

Despite the contributions of the present study, several limitations warrant consideration. First, the sample consisted predominantly of college students, which may constrain the generalizability of the findings to broader age groups or populations with different educational backgrounds. Second, several relevant variables were not measured in the current design. Factors such as the participants’ age, religious beliefs, and specific moral emotions (e.g., guilt, shame, empathy) could have significantly influenced their moral decision-making processes. Their omission limits the depth of interpretation and highlights the need for more comprehensive assessments in future research.

Building on these limitations, future studies should pursue several concrete directions. One promising avenue involves examining the role of moral emotions—particularly guilt and shame—in shaping decisions under moral uncertainty. Such investigations could clarify the emotional mechanisms underlying moral reasoning. Additionally, cross-cultural replications are essential to explore how cultural norms and values modulate the interaction between emotion and cognition in moral judgment. Finally, longitudinal designs could provide insight into how moral reasoning evolves across developmental stages, offering a richer understanding of the temporal dynamics of moral cognition.

## 7. Conclusions

This research demonstrates that moral judgment is deeply embedded within social and cultural contexts. From the three studies conducted here, we find that individuals’ moral evaluations are significantly influenced by the nature of their relationship with the parties involved, supporting the predictions of Relationship Regulation Theory. By adopting a first-person perspective and by comparing Chinese and American participants, our findings reveal that relational context plays a crucial role in shaping moral priorities and that these priorities vary across cultures. These insights underscore the importance of considering both interpersonal dynamics and cultural background when examining moral cognition, offering a more nuanced understanding of how people navigate ethical decisions in everyday life.

## Figures and Tables

**Table 1:** Mean and standard deviations of moral judgments for each relational context.

Social Relationship	Moral Judgements	
	*M*	*SD*
Parents	4.86	0.99
Superiors	5.25	1.17
Colleagues	5.06	1.12
Salesperson	5.50	0.97

**Table 2:** Mean wrongness judgment for each relational context and moral-foundation-set of violations.

	**Moral Foundation Being Violated**				
Relational Context	Care	Fairness	Loyalty	Respect	Purity
	*M* (*SD*)				
Parents	5.17 (0.74)	4.76 (0.78)	5.36 (0.74)	5.57 (0.89)	5.99 (0.84)
Teacher	5.49 (0.68)	5.15 (0.74)	5.21 (0.78)	5.84 (0.78)	6.31 (0.67)
Colleagues	5.28 (0.63)	5.14 (0.70)	5.11 (0.77)	5.60 (0.80)	5.88 (0.83)
Salesperson	5.34 (0.66)	5.29 (0.71)	4.83 (0.80)	5.73 (0.81)	6.21 (0.80)

## Data Availability

Data will be provided by the authors upon request.
